# The Big Three Health Behaviors and Mental Health and Well-Being Among Young Adults: A Cross-Sectional Investigation of Sleep, Exercise, and Diet

**DOI:** 10.3389/fpsyg.2020.579205

**Published:** 2020-12-10

**Authors:** Shay-Ruby Wickham, Natasha A. Amarasekara, Adam Bartonicek, Tamlin S. Conner

**Affiliations:** Department of Psychology, University of Otago, Dunedin, New Zealand

**Keywords:** sleep, health behaviors, physical activity, diet, sleep quality, mental health, well-being, sleep quantity

## Abstract

**Background:**

Sleep, physical activity, and diet have been associated with mental health and well-being individually in young adults. However, which of these “big three” health behaviors most strongly predicts mental health and well-being, and their higher-order relationships in predictive models, is less known. This study investigated the differential and higher-order associations between sleep, physical activity, and dietary factors as predictors of mental health and well-being in young adults.

**Method:**

In a cross-sectional survey design, 1,111 young adults (28.4% men) ages 18–25 from New Zealand and the United States answered an online survey measuring typical sleep quantity and quality; physical activity; and consumption of raw and processed fruit and vegetables, fast food, sweets, and soda, along with extensive covariates (including demographics, socioeconomic status, body mass index, alcohol use, smoking, and health conditions) and the outcome measures of depressive symptoms [measured by the Center for Epidemiological Depression Scale (CES-D)] and well-being (measured by the Flourishing Scale).

**Results:**

Controlling for covariates, sleep quality was the strongest predictor of depressive symptoms and well-being, followed by sleep quantity and physical activity. Only one dietary factor—raw fruit and vegetable consumption—predicted greater well-being but not depressive symptoms when controlling for covariates. There were some higher-order interactions among health behaviors in predicting the outcomes, but these did not survive cross-validation.

**Conclusion:**

Sleep quality is an important predictor of mental health and well-being in young adults, whereas physical activity and diet are secondary but still significant factors. Although strictly correlational, these patterns suggest that future interventions could prioritize sleep quality to maximize mental health and well-being in young adults.

## Introduction

Healthy lifestyles are important contributors to both physical and mental health. Getting high-quality sleep, engaging in physical activity, and eating well not only have advantages to physical health (Chin et al., 2019) but also have advantages to mental health such as reduced risk of depression (Raboch et al., 2017; Francis et al., 2019) and anxiety (Nho and Yoo, 2018) and increased psychological well-being (Pilcher et al., 1997; Mujcic and Oswald, 2016; Prendergast et al., 2016; Conner et al., 2017). Healthy lifestyles may be especially important for the mental health and well-being of young adults. Emerging adulthood is a time of both developmental and ecological changes, marked by increased responsibility, new roles, and changing life circumstances (Conley et al., 2020). This developmental period often coincides with a transition to work or university, with changing routines, academic demands, and living situations, which can disrupt health behaviors (Conley et al., 2014). Emerging adults also appear more vulnerable to poorer mental health (Adolescent Health Research Group, 2008; Stallman, 2010), which could suggest a role for unhealthy lifestyles contributing to poorer emotional functioning.

There is good evidence linking the “big three” health behaviors of sleep, physical activity, and diet individually to both mental health and well-being in emerging adults. Sleep is one modifiable health behavior that may become particularly disrupted in this population (Lev Ari and Shulman, 2012). Sleep plays a vital role in both mental and physical health across the lifespan, with approximately one third of each day dedicated to sleep (Samson and Nunn, 2015). It is recommended that healthy adults need approximately 7–8 h sleep per night, while healthy emerging adults need approximately 7–9 h sleep per night (Hirshkowitz et al., 2015). Both inadequate and disrupted sleep has been shown to negatively influence mental and physical health and are risk factors for both depression and anxiety (Alfano and Gamble, 2009; Harvey, 2011). However, while sleep quantity is associated with increased depression and negative affect among clinical populations, sleep quality appears to be a greater predictor of mental health and well-being among the general population (Pilcher et al., 1997; Bassett et al., 2015; Wallace et al., 2017; João et al., 2018) and young adults in particular (Buysse et al., 2008). Furthermore, while clear recommendations around sleep quantity are outlined and promoted, the importance of sleep quality in mental health and well-being receives less attention.

Physical activity is the second modifiable health behavior tied to better mental health and well-being in young adults. Physical activity releases endorphins within the body, which help to promote well-being and feelings of euphoria, and increase mood and energy (Fox, 1999). A recent meta-analysis of 16 randomized control trials (RCTs) suggests that regular physical activity at a moderate intensity may aid in the treatment of mental disorders such as depression (Bailey et al., 2018). Additionally, physical activity has been associated with improved well-being among non-clinical young adult populations (Penedo and Dahn, 2005) and associated with happiness more generally (Zhang and Chen, 2019). Conversely, low levels of physical activity and increased sedentary behavior have been associated with poorer mental well-being among adolescents (Ussher et al., 2007).

Diet is the third modifiable lifestyle behavior that contributes to mental health and well-being in young adults. Research has shown regular adherence to a healthy diet is associated with reduced risk of depression and improved mood (Molendijk et al., 2018). Intake of fruits and vegetables is a key aspect of a healthy diet linked to greater happiness and well-being in young adults (White et al., 2013; Conner et al., 2015), with further evidence that consumption of raw fruits and vegetables may be more beneficial than consumption of cooked or processed fruits and vegetables (Brookie et al., 2018). Conversely, regular consumption of a typical Western diet, categorized by consumption of refined grains, high sugar intake, and processed and fried foods, has been associated with increased perceived stress among female college students, increased depressive symptoms among both male and female students (El Ansari et al., 2014), poorer mental health outcomes (Jacka et al., 2010), and increased risk of depression (Akbaraly et al., 2009).

While extensive research has shown the mental health and well-being benefits of sleep, physical activity, and diet as individual predictors, research examining all three behaviors together, along with their possible higher-order relationships, is limited. Prior research has shown possible synergistic relationships between health behaviors in predicting well-being (Prendergast et al., 2016), which warrants replication. However, compensatory relationships could also be found, whereby the Compensatory Carry-Over Action Model suggests a good diet could compensate for low physical activity (Tan et al., 2018). Knowing the importance of each of these lifestyle behaviors, singularly or in combination with each other, and the hierarchical order of importance will inform mental health interventions at both the population and individual level.

Therefore, the current study investigated how self-reported sleep, physical activity, and dietary factors together predicted differences in mental health and well-being in young adults and whether there were any higher-order interactions among these behaviors in the prediction patterns.

## Methods

### Ethics Statement

The “Lifestyles of Young Adults” study was approved by the University of Otago Department of Psychology (Category B Ethics #D17/158), with oversight by the University of Otago Ethics Committee.

### Design and Participants

We used a cross-sectional correlational design with data collected through an online survey and extensive measurement of demographic and health covariates. Participants were 1,111 young adults (316 men, 28.4%) between 18 and 25 years old (*M* = 22.02, *SD* = 2.34) living in New Zealand or the United States. The New Zealand participants were recruited through a psychology department experiment participation program and reimbursed with extra course credit. The United States participants were recruited using Amazon’s Mechanical Turk (MTurk) to broaden the diversity of our sample and reimbursed with US$1.50 (MTurk; *n* = 784). Recruitment and data collection occurred during April and May of 2018 (*n* = 354) and 2019 (*n* = 757). Data collected from 2017 (*n* = 422) was previously published in Brookie et al. (2018), with no overlap in data with the present paper. Participants were required to be 18–25 years old at the time of participation. Inclusion criteria specific to MTurk participants were as follows: living in the United States, passing attention checks dispersed throughout the survey, having a Human Intelligence Task (HIT) approval rate > 90%, and having not done the survey in previous years.

### Survey Measures

#### Health Behaviors

Sleep quantity was assessed by asking “In a typical week, how many hours do you usually sleep per night?” (0–20 h) (adapted from the Basic Nordic Sleep Questionnaire, Partinen and Gislason, 1995, where the original question asked “How many hours do you usually sleep per night? I sleep about ____ hours per night.”). Sleep quality was assessed with a non-validated question by asking “When you wake up from sleeping, how refreshed do you feel?” (0) “Never refreshed,” (1) “A little or somewhat refreshed,” (2) “Moderately refreshed,” (3) “Mostly refreshed,” (4) “Very refreshed,” because refreshed sleep is a key indicator of high-quality sleep (Libman et al., 2016).

Physical activity was assessed using a single item by Milton et al. (2011): “In a typical week, on how many days have you done a total of 30 min or more of physical activity, which was enough to raise your breathing rate? This may include sport, exercise, and brisk walking or cycling for recreation or to get to and from places, but should not include housework or physical activity that is part of your job” (0–7 days a week).

*Diet* used items modified from the New Zealand Nutrition Survey and adapted from similar research by Mujcic and Oswald (2016) and Lesani et al. (2016). Participants rated their typical consumption in a week of raw fruit, raw vegetables, processed (cooked, frozen, or canned) fruit, processed vegetables, fast food, sweets, and soda consumption via two questions per food group, for example, (1) “How many days in a typical week do you consume raw fruit?” (0–7 days/week) and (2) “On a day when you consume raw fruit, how many servings do you have?” (0–7 + servings), if Q1 was 1–7 days. Examples of the foods and serving sizes were provided (details in Brookie et al., 2018).

#### Mental Health and Well-Being

Our measure of mental health was depressive symptoms using the Center for Epidemiological Depression Scale (CES-D; Radloff, 1977). This scale contained 20 items related to feelings “In the last week, including today.” Examples included “I was bothered by things that usually don’t bother me” and “I felt depressed,” answered on a four-point scale (0) “Rare or none of the time (<1 day),” (1) “Some or a little of the time (1–2 days),” (2) “Occasionally or a moderate amount of the time (3–4 days),” (3) “Most or all of the time (5–7 days).” After reverse scoring and imputing missing values, items were summed (α = 0.854).

Our measure of well-being was flourishing using the Flourishing Scale (Diener et al., 2010). This scale contained eight statements such as “I lead a purposeful and meaningful life” and “I am engaged and interested in my daily activities,” rated on a seven-point Likert scale (1) strongly disagree to (7) strongly agree, which were averaged (α = 0.918).

#### Demographic and Health Covariates

*Demographic covariates* included age (18–25 years), gender (male, female, or gender diverse), ethnicity (e.g., Caucasian/White, African-American/Black, Asian, Hispanic, or mixed/other), education level, employment status, and both childhood and adult socioeconomic status (SES) status (Griskevicius et al., 2011). Health covariates included body mass index (BMI) measured through questions about height and weight, with response options available in both metric and imperial units. Health conditions were measured by a series of tick boxes to indicate presence of 12 common conditions (e.g., diabetes, hypertension, celiac disease, disordered eating behaviors, anemia, etc., or another condition not listed). Participants were also asked about any food allergies, whether they excluded meat and/or animal products from their diet (vegetarian status), alcohol consumption (days in a typical week they consume alcohol and standard drinks typically consumed on drinking days), and smoking frequency, along with current antidepressant medication use and vitamin use.

### Data Preparation and Analyses

From an original sample of 1,522 participants, 411 were excluded for failing to pass attention checks (*n* = 370), having incomplete data (*n* = 23), displaying a response bias (*n* = 7), or missing key variables (*n* = 11), resulting in a final sample of 1,111 participants. Overall SES was computed by averaging childhood and adult SES (α = 0.809). BMI was computed by dividing weight in kilograms by the square of height in meters (Nuttall, 2015). For each food category, an average daily food serving was calculated by multiplying days per week × servings and dividing by seven (e.g., raw fruit 4 days per week × two servings = eight weekly servings/7 days = 1.14 daily servings). Daily servings of raw fruit and raw vegetables were summed for a raw fruits and vegetables (FV) variable; processed fruit and processed vegetables were summed for a processed FV variable. Daily servings of chocolate and candy were summed for a sweets variable. Average daily alcohol consumption was calculated by multiplying days they consume alcohol per week × standard drinks and dividing by seven. Ethnicity was coded using four dummy codes with White ethnicity as the reference group (vs. Asian, Black, Hispanic, mixed/other). Gender was coded using two dummy codes with male gender as the reference group (vs. Female, Gender Diverse). Employment status was dummy coded with those who were employed or studying as the reference group (0) vs. those who were unemployed (1). Smoking was dummy coded as non/infrequent smokers (0) versus regular smokers (1) who smoked once/week or more. Health condition, antidepressant use, food allergy, and vegetarian status were each dummy coded to indicate absence (0) or presence (1). Descriptive statistics and bivariate correlations among the health behaviors and depressive symptoms and flourishing were computed. All continuous variables were centered prior to analysis.

Hierarchical regression analyses were run to predict depressive symptoms and flourishing as separate outcomes from sleep quantity, sleep quality, physical activity, raw FV, processed FV, fast food, sweets, and soda, as simultaneous predictors, controlling for the demographic and health covariates. Quadratic factors for the sleep, activity, and diet variables were included to test for any non-linear associations with the outcomes and were retained only when significant. Covariates were included in the model if they correlated with either the predictors and/or the outcome measures. Model 1 included only the covariates; model 2 added the health behaviors as simultaneous predictors plus the significant quadratic terms; model 3 added the significant two-way interaction terms among the health behaviors. Analyses predicting depressive symptoms were repeated excluding the one item from the CES-D that measured sleep quality (“My sleep was restless”) to ensure that any association between self-reported sleep quality and depressive symptoms was not an artifact of overlapping measurement. We also used 10-fold cross-validation to determine whether any interaction terms would be useful for predicting out-of-sample, above and beyond the no-interaction model. Cross-validation involves splitting data into several subsets or “folds” and then repeatedly fitting the model to all but onefold and testing the model on the leftover fold (Koul et al., 2018).

## Results

Tables 1, 2 present the participant characteristics and descriptive statistics for all measures. The majority of participants were free of health conditions (69.8%), with 20% using antidepressants, 6.8% identifying as vegetarian/vegan, 16.6% having a food allergy, and 8.5% smoking regularly. Sixty-one percent of participants were currently attending college (60.8%) with roughly equal numbers of college-attending students from New Zealand (*n* = 327) and the United States (*n* = 348 MTurk workers). Participants’ average BMI score of 22.93 was within the normal range (18.5–24.9). On average, participants slept approximately 7 h per night, rated their sleep quality around the “a little or somewhat refreshed” level (1.63), and engaged in physical activity approximately 3 days per week (2.94). Participants ate an average of approximately three servings of fruit and vegetables per day (3.10), below the recommended five servings per day (Rekhy and McConchie, 2014), but consistent with previous research within the young adult population (e.g., Laska et al., 2012). The sample showed elevated depressive symptoms, with an average CES-D score of 19, where scores above 16 indicate risk of clinical depression. The overall well-being of the sample was slightly positive, with the average flourishing score of five (corresponding with *slightly agree*). The bivariate correlations among the health behaviors and with depressive symptoms and flourishing are presented in Supplementary Tables 1, 2. The health behaviors were weakly correlated with each other and most were significantly correlated with depressive symptoms and flourishing. The correlation between depressive symptoms and flourishing was *r* = −0.707, *p* < 0.001. The bivariate correlations among the demographic factors and the health behaviors are presented in Supplementary Table 3.

**TABLE 1:** Descriptive statistics for categorical participant characteristics.

**Categorical measures**	**Categories**	***n* (% of sample)**
Gender	Female	776 (69.8)
	Male	316 (28.4)
	Gender diverse	19 (1.7)
Ethnicity (top 6)	White	727 (65.4)
	Mixed	121 10.9)
	Asian	87 (7.8)
	Other	72 (6.5)
	Black	60 (5.4)
	Hispanic	44 (4.0)
Sample	MTurk	784 (70.6)
	Psychology students	327 (29.4)
Year	2018	354 (31.9)
	2019	757 (68.1)
College	Current college student	675 (60.8)
	Not a current college student	436 (39.2)
Education	Attended tertiary undergrad	576 (51.8)
	Completed tertiary undergrad	224 (20.4)
	Attending tertiary higher	88 (7.9)
	Completed tertiary higher	86 (7.7)
	Did not complete high school	4 (0.4)
	Completed high school	133 (12.0)
Employment	Full or part-time work	566 (51.0)
	Full or part-time student	681 (61.3)
	Unemployed	102 (9.2)
Health condition	Any health condition	336 (30.2)
Antidepressant use	Yes	222 (20.0)
Supplement use	Yes	487 (43.8)
Food exclusion	Vegetarian/vegan	75 (6.8)
	Food allergy	184 (16.6)
Smoker	Yes	94 (8.5)

*“Sample” is identical to country.*

**TABLE 2:** Descriptive statistics for continuous participant characteristics, health behaviors, and psychological outcomes.

**Continuous measures**	**Mean (*SD*)**	**Range**
Age	22.02 (2.34)	18.00–25.00
SES (combined)	3.99 (1.30)	1.00–7.00
BMI	22.93 (6.19)	11.05–57.81
Alcohol (standard drinks/day)	0.74 (1.19)	0.00–12.00
Sleep quantity (hours/night)	7.21 (1.36)	2.00–20.00
Sleep quality	1.63 (1.00)	0.00–4.00
Physical activity (days/week)	2.94 (1.95)	0.00–7.00
Raw FV (servings/day)	2.03 (1.75)	0.00–14.00
Processed FV (servings/day)	1.07 (1.03)	0.00–8.57
Fast food (servings/day)	0.27 (0.39)	0.00–5.00
Sweets (servings/day)	0.71 (0.94)	0.00–7.86
Soda (servings/day)	0.51 (1.00)	0.00–7.00
Depressive symptoms	19.45 (13.00)	0.00–58.00
Flourishing	5.01 (1.20)	1.00–7.00

*BMI, body mass index; SES, socioeconomic status from childhood and adulthood combined; FV, fruit and vegetable consumption. Depressive symptoms were measured with the Center for Epidemiological Studies Depression Scale (CES-D) with an absolute range of 0–60; scores of 16 or higher are considered indicative of depression. Flourishing was measured using the Flourishing Scale, with an absolute range of 1–7.*

**FIGURE 1:**
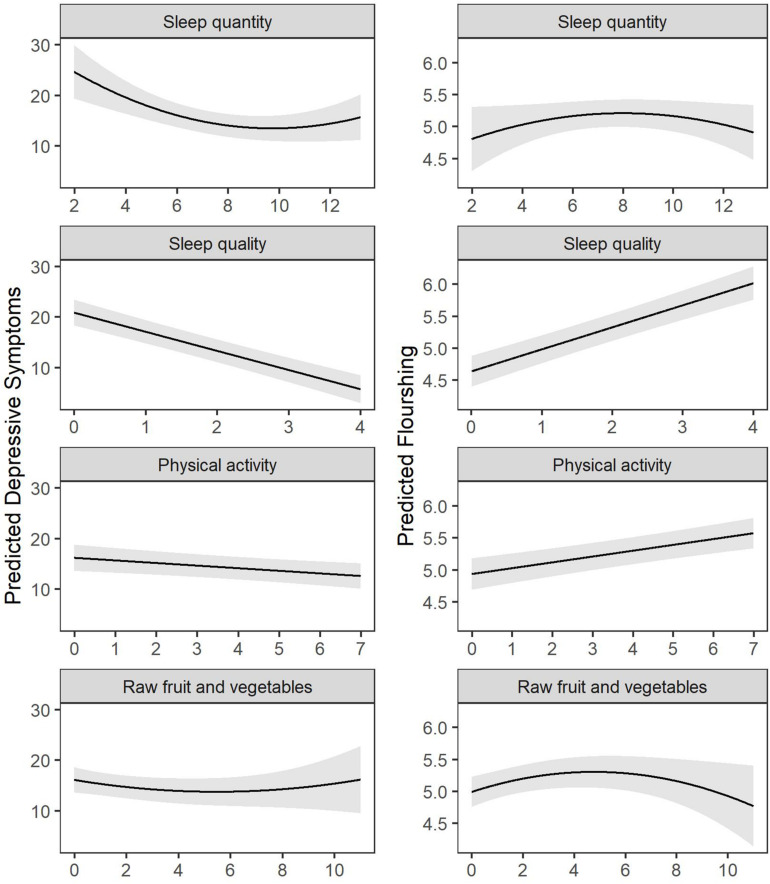
Visualization of hierarchical regression model results predicting depressive symptoms and flourishing, adjusted for other health behaviors and covariates (*n* = 1,111).

The regression results are presented in Table 3 and visualized in Figure 1. As indicated in Table 3 Model 2, when controlling for the demographic and health covariates, sleep quantity and quality were the strongest lifestyle predictors of depressive symptoms. Individuals who slept inside the range of 8–12 h per night (not more or less) and who had better sleep quality reported fewer depressive symptoms. Physical activity was the second strongest predictor of depressive symptoms, and, similar to sleep quality, had a linear but weaker dose-response relationship with depressive symptoms. Unexpectedly, dietary factors did not predict depressive symptoms in the controlled regression model. Importantly, sleep quality and the other health behaviors continued to predict depressive symptoms to the same degree when removing the one item from the CES-D measuring restless sleep (sleep quality predicting modified CES-D; *B* = −0.282, *p* < 0.001 vs. unmodified CES-D; *B* = −0.293, *p* < 0.001). Patterns when predicting flourishing were similar but not identical. For flourishing, sleep quality was the outperforming predictor, followed by physical activity, then raw FV intake, then sleep quantity (quadratic only). Raw FV had both a linear and curvilinear relationship to flourishing, whereby greater quantities of raw FV after eight servings per day no longer predicted any further well-being. The lifestyle behaviors accounted for 11.5% of the variance in depressive symptoms and 12.0% of the variance in flourishing. All of the variables together accounted for 38.0% of the variance in depressive symptoms and 35.4% of the variance in flourishing. Residuals from all multivariate models were normally distributed, indicating the assumptions of normality underlying regression models were met.

**TABLE 3:** Hierarchical regression models predicting depressive symptoms and flourishing (*n* = 1,111).

	**Depressive symptoms *B*, *b* (SE)**	**Flourishing *B*, *b* (SE)**
**Model 1—only covariates^a^**		
*R*^2^ change	0.265***	0.234***
*F* change (df)	21.901 (18, 1,092)	18.578 (18, 1,092)
**Model 2—adding health behaviors**		
*Intercept*	n/a, 5.317 (3.266)	n/a, 5.211 (0.108)***
***Covariates***		
Age	−0.113, −0.625 (0.199)**	0.074, 0.038 (0.019)*
Gender—Female	0.039, 1.113 (0.754)	0.073, 0.190 (0.071)**
Gender—Diverse	0.039, 3.906 (2.565)	0.028, 0.257 (0.242)
Ethnicity—Asian	0.038, 1.827 (1.231)	−0.068, −0.304 (0.116)**
Ethnicity—Black	0.009, 0.495 (1.454)	0.043, 0.228 (0.137)
Ethnicity—Hispanic	0.055, 3.641 (1.641)*	−0.045, −0.275 (0.155)
Ethnicity—mixed/other	0.021, 0.730 (0.866)	−0.007, −0.024 (0.082)
Sample	0.026, 0.734 (1.112)	−0.097, −0.256 (0.105)*
Unemployment	0.035, 1.596 (1.122)	−0.070, −0.292 (0.106)**
SES	−0.227, −2.263 (0.270)***	0.264, 0.244 (0.026)**
BMI	0.039, 0.082 (0.055)	−0.004, −0.001 (0.005)
Health condition	0.141, 3.991 (0.741)***	−0.038, −0.100 (0.070)
Antidepressant use	0.112, 3.628 (0.833)***	−0.066, −0.198 (0.079)*
Supplement use	0.002, 0.033 (0.400)	0.008, 0.012 (0.038)
Food allergy	0.026, 0.900 (0.883)	0.020, 0.064 (0.084)
Vegetarian	0.012, 0.626 (1.285)	−0.001, −0.005 (0.121)
Alcohol	−0.001, −0.006 (0.285)	0.008, 0.008 (0.027)
Smoking	0.012, 0.563 (1.175)	0.038, 0.166 (0.111)
***Health behaviors***		
Sleep quantity	−0.379, −3.630 (0.970)***	0.020, 0.018 (0.025)
Sleep quantity quadratic	0.321, 0.186 (0.058)**	−0.056, −0.011 (0.005)*
Sleep quality	−0.293, −3.788 (0.357)***	0.287, 0.344 (0.034)***
Physical activity	−0.079, −0.526 (0.180)**	0.147, 0.091 (0.017)***
Raw FV	−0.036, −0.270 (0.210)	0.109, 0.075 (0.027)**
Raw FV quadratic	−	−0.092, −0.014 (0.005)**
Processed FV	0.048, 0.602 (0.330)	−0.052, −0.060 (0.031)
Fast food	0.017, 0.560 (0.915)	−0.049, −0.151 (0.086)
Sweets	0.048, 0.661 (0.359)	−0.003, −0.004 (0.034)
Soda	0.012, 0.156 (0.337)	−0.025, −0.030 (0.032)
*R*^2^ change for health Bx	0.115***	0.120***
*F* change (df) for health Bx	22.286 (9, 1,083)	20.045 (10, 1,082)
*Cross-validated R^2^*	0.308 (0.073)	0.308 (0.073)
*Overall R^2^*	0.380	0.354

“Sample” is identical to country. B, standardized regression coefficient; b, unstandardized regression coefficient; BMI, body mass index; Bx, behaviors; FV, fruits and vegetables; SE, standard error; SES, socioeconomic status from childhood and adulthood combined; continuous measures were all centered. Sample was coded 0 for psychology and 1 for MTurk. *p < 0.05; **p < 0.01; ***p < 0.001. ^*a*^Model 1 included just the 18 covariate variables from age to smoking (see Supplementary Table 5 for full model 1 results).

Finally, analyses revealed several significant two-way interactions between the health behaviors in predicting depressive symptoms and flourishing (Supplementary Table 5). The first two patterns indicated a mild compensatory relationship, whereby eating more raw FV compensated for low physical activity in both depressive symptoms and flourishing. The second pattern indicated a mild compensatory relationship, whereby eating more raw FV partially compensated for poor sleep quantity in flourishing only. However, these interactions did not survive the cross-validation procedure, suggesting they are not replicable (Supplementary Material).

## Discussion

Sleep quality may be the most important health behavior predicting mental health and well-being in young adults, more so than sleep quantity, physical activity, and dietary factors such as raw fruit and vegetable intake. Although our study was only a correlational design, the patterns were robust when controlling for demographic and health covariates and are consistent with previous research (Bassett et al., 2015; Di Benedetto et al., 2020). The findings from our study add to this literature by showing that when tested side-by-side, sleep quality significantly outranked other health behaviors in the prediction of mental health and well-being. Moreover, there was a significant quadratic relationship between sleep quantity and both depressive symptoms and flourishing, such that too little sleep (<8 h) and too much sleep (>12 h) were associated with higher depressive symptoms and lower flourishing. Depressive symptoms were lowest for young adults who slept 9.7 h per night and flourishing was highest for young adults who slept 8 h per night. This finding is consistent with previous research showing a U-shape relationship between sleep quantity and mental health among Japanese adolescents (Kaneita et al., 2007) and suggests this phenomenon is pervasive across geographical and cultural environments.

When considering the other health behaviors, physical activity was also a significant predictor of depressive symptoms and flourishing, but, descriptively, it was not strong as sleep quality. These findings are consistent with previous research suggesting that physical activity can contribute to greater mental health (Ghrouz et al., 2019) and well-being (Costigan et al., 2019; Zhang and Chen, 2019), providing support for physical activity interventions to improve mental health and well-being.

Consumption of raw FV but not processed FV significantly predicted flourishing. Flourishing was highest for young adults who ate 4.8 servings of raw FV per day, and, similar to sleep quantity, too little raw FV (< two servings) or too high raw FV (> eight servings) were associated with lower flourishing. This result replicates previous research showing that raw rather than cooked or processed FV may be more beneficial to well-being and that excessive intake may be problematic (Brookie et al., 2018). However, contradictory to previous research, raw FV did not predict lower depressive symptoms in our controlled models, which may be due to the shared variance with physical activity which outperformed raw FV in predicting depressive symptoms. Similarly, unhealthy food consumption did not significantly predict mental health or well-being in our controlled models. This may be due to the relatively low consumption of unhealthy foods in the present sample, our narrow measurement of unhealthy foods, or possible covariation with other factors that played a stronger role.

The present study tested for higher-order relationships among the health behaviors and found minimal evidence. Although our regression results revealed three significant interactions, whereby consumption of raw FV appeared to mitigate some of the negative effects of having poor quantity sleep or limited exercise, these interactions were not significant when tested with cross-validation models. This lack of validation suggests these interactions are not especially strong or replicable.

The results of this study hold practical significance, supporting and extending previous intervention research showing that engaging in one or more health behaviors can enhance mental health and well-being. Young adults have been identified as a population at risk of elevated mental health disorders, with extremely high reports of mental health problems and psychological distress (Stallman, 2010; Johnston et al., 2014). The present study supported this finding, with an average depression score of 19 on the CES-D, where scores over 16 correspond to increased risk of clinical depression. Therefore, health promotion interventions targeting sleep, diet, and exercise within young adult populations may help to promote optimal mental health and well-being within this at-risk population, as targeting one individual behavior may not always improve other health behaviors.

There were several strengths of this study. We had a large sample of college-attending and non-college-attending young adults, encompassing two geographic locations (New Zealand and United States). The breadth of our sample suggests the phenomenon of young adult mental ill-health is pervasive and does not necessarily reflect geographical or cultural environments. Furthermore, this study examined multiple health behaviors simultaneously, including sleep, along with the typical measures of healthy diet and physical activity.

However, as this study is a correlational design, we cannot be certain that the health behaviors are driving the differences in mental health and well-being. For example, it is possible that depressive symptoms could be causing sleep disturbances or that greater well-being could increase engagement in positive health behaviors (Fredrickson et al., 2020). However, evidence shows that disrupted sleep more often precedes the onset of depression in young adults (Buysse et al., 2008) and that intervening to change health behaviors results in improvements to mental health and well-being (Carr et al., 2013; Conner et al., 2017; Jacka et al., 2017; Francis et al., 2019). Our study relied on participants’ accurate recall of their health and lifestyle behaviors on a typical week, which could lead to inaccurate reporting. Despite this, the present study found behaviors reported by the young adults to be consistent with that of previous research among this population (Parker et al., 2007; Laska et al., 2012), suggesting that this data collection method may be sufficient, although not preferred. Follow-up research using objective measures is warranted (e.g., using accelerometer data to track hours of sleep and physical activity). Another limitation is the single-item sleep and physical activity questionnaires. We used these shortened measures to reduce the burden on participants because the survey was long. However, future research should consider using the full Basic Nordic Sleep Questionnaire (Partinen and Gislason, 1995) or similar measures such as the Pittsburgh Sleep Quality Index (Buysse et al., 1989) to measure other sleep parameters linked to mental health. Similarly, more extensive exercise measures could be used to determine how other factors aside from frequency of exercise, such as intensity and duration, might be related to mental health and well-being. Finally, the regression models used in this analysis controlled for the shared variance between sleep, physical activity, and diet; any shared variance between them was excluded from the multiple regression analyses. As such, this study has only shown the unique contributions of each health behavior. However, as indicated previously, the correlations between the health behaviors were relatively low. To address this issue of shared variance, future research examining clusters of health behaviors should consider using more sophisticated analyses that model the underlying common variance, possibly as an additional predictor.

## Conclusion

This study highlighted the relative contribution of sleep, physical activity, and diet to the prediction of depressive symptoms and flourishing. Our findings suggest that future lifestyle interventions targeting sleep quality may be most beneficial at improving mental health and well-being. However, physical activity and diet should not be disregarded, particularly as they also uniquely predicted differences in depressive symptoms (physical activity) and well-being (physical activity and raw fruit and vegetable intake). Sleep, physical activity, and a healthy diet should be thought of as multiple tools for promoting optimal mental health and well-being, particularly among young adult populations where the prevalence of mental disorders is high and well-being is suboptimal.

## Data Availability Statement

The datasets presented in this study can be found in online repositories. The names of the repository/repositories and accession number(s) can be found below: If you would like to use these data for research purposes, please ask permission by emailing the owner, TC (tamlin.conner@otago.ac.nz), to make sure that your research question is not duplicating ongoing work. Explicit permission by and co-authorship with the owner is required for using these data for publication or presentation purposes. The dataset analyzed for this study can be found in the Open Science Framework (OSF) Repository https://osf.io/pdnce/.

## Ethics Statement

The studies involving human participants were reviewed and approved by the University of Otago Department of Psychology (Category B Ethics #D17/158), with oversight by the University of Otago Ethics Committee. The patients/participants provided their written informed consent to participate in this study.

## Author Contributions

S-RW conceived the idea, collected and analyzed data, and co-wrote the manuscript. NA conceived the idea and collected data. AB co-wrote the manuscript and visualized data. TC conceived the idea, co-wrote the manuscript, and provided supervisory support to S-RW, NA, and AB. All authors contributed to the article and approved the submitted version.

## Conflict of Interest

The authors declare that the research was conducted in the absence of any commercial or financial relationships that could be construed as a potential conflict of interest.
