# Supplementary material for: The Big Three Health Behaviors and Mental Health and Well-Being Among Young Adults: A Cross-Sectional Investigation of Sleep, Exercise, and Diet
Source: Front Psychol. 2020 Dec 10;11:579205. doi: 10.3389/fpsyg.2020.579205 (PMC7758199; doi:10.3389/fpsyg.2020.579205)
Supplement: Supplementary file 1 [file Data_Sheet_1.pdf]

Supplementary Material

Supplementary Table 1

Bivariate Correlations among Health Behaviors (N=1111)

	Sleep Quality	Physical Activity	Raw FV	Processed FV	Fast Food	Sweets	Soda
Sleep Quantity	0.245***	-0.015	0.009	-0.002	-0.016	-0.033	-0.028
Sleep Quality	-	0.159***	0.142***	0.012	-0.096**	-0.097**	-0.053
Physical Activity		-	0.374***	0.192***	-0.081**	0.009	-0.151***
Raw FV			-	0.339***	-0.092**	0.030	-0.127***
Processed FV				-	0.033	0.156***	-0.027
Fast Food					-	0.286***	0.248***
Sweets						-	0.105***

Note. ***p* < .01, ****p* < .001; FV = Fruit and Vegetable consumption; Sweets = Chocolate and Candy combined

Supplementary Table 2

Bivariate Correlations between Health Behaviors and Depressive Symptoms / Flourishing (n = 1111)

	Depressive Symptoms	Flourishing
Sleep Quantity	-0.178***	0.100**
Sleep Quality	-0.475***	0.415***
Physical Activity	-0.198***	0.279***
Raw FV	-0.136***	0.212***
Processed FV	0.019	0.038
Fast Food	0.136***	-0.161***
Sweets	0.118***	-0.046
Soda	0.111***	-0.140***

Note. ****p* < .001; Correlations are not adjusted for any covariates.
FV = Fruit and Vegetable consumption; Sweets = Chocolate and Candy combined.

Supplementary Table 3

Bivariate Correlations between Covariates and Health Behaviors (n = 1111)

Covariate	Sleep Quantity	Sleep Quality	Physical Activity	Raw FV	Processed FV	Fast Food	Sweets	Soda
Age	-0.046	0.039	-0.100**	-0.137***	-0.072*	0.070*	-0.055	0.129***
Gender[1]	-0.093**	-0.168***	-0.057	0.066*	0.054	-0.119***	0.070*	-0.093**
Ethnicity[2]	-0.067*	0.021	-0.040	-0.053	0.009	0.120***	0.025	-0.060*
Sample[3]	-0.043	0.056	-0.163***	-0.180***	-0.142***	0.102**	-0.110***	0.186***
Unemployment[4]	0.020	-0.073*	-0.096**	-0.096**	-0.026	-0.004	0.018	0.052
SES	0.136***	0.303***	0.189***	0.189***	0.071*	-0.144***	-0.038	-0.120***
BMI	-0.097**	-0.132***	-0.180***	-0.111***	-0.103**	0.163***	-0.038	0.203***
Health Condition[5]	-0.024	-0.199***	-0.036	0.013	0.032	0.045	0.072*	0.095**
Antidepressant use[6]	0.026	-0.111***	-0.114***	-0.066*	-0.003	0.023	-0.026	0.109***
Supplement use[7]	0.002	0.015	0.047	0.149***	0.051	-0.014	0.041	-0.067*
Food Allergy[8]	-0.015	-0.054	0.025	0.080**	0.073*	0.102**	0.093**	0.011
Vegetarian[9]	-0.004	-0.048	0.016	0.151***	0.115***	-0.086**	0.009	-0.055
Alcohol	0.021	-0.074*	0.035	0.051	0.050	0.115***	0.065*	-0.009
Smoking[10]	-0.004	-0.037	-0.062*	-0.010	0.063*	0.147***	0.083**	0.150**

Note. *$p < .05$, **$p < .01$, ***$p < .001$. SES = socioeconomic status; BMI = body mass index

[1] male (0) vs. female/gender diverse (1); [2] white (0) vs. non-white /mixed ethnicity (1); [3] psychology (0) vs. MTurk (1); [4] not unemployed (0) vs. unemployed (1); [5] none (0) vs. any current medical condition (1); [6] not currently taking (0) vs. currently taking antidepressant/mood stabilizing medication (1); [7] not currently taking (0) vs. currently taking vitamin supplements (1); [8] no food allergy (0) vs. food allergy (1); [9] non-vegetarian (0) vs. vegetarian/vegan (1); [10] non-smoker (0) vs. smoker (1).

Supplementary Table 4

Hierarchical Regression Model 1 using only the Covariates to Predict Depressive Symptoms and Flourishing (n = 1111).

Model 1- only covariates	Depressive Symptoms<br>B, b (SE)	Flourishing<br>B, b (SE)
Intercept	-n/a, 13.891 (1.216) ***	-n/a, 5.306 (0.115) ***
Covariates		
Age	-0.126, -0.699 (0.214) **	0.093, 0.048 (0.020) *
Gender - Female	0.080, 2.263 (0.798) **	0.039, 0.102 (0.075)
Gender - Diverse	0.054, 5.410 (2.759) *	0.003, 0.029 (0.261)
Ethnicity - Asian	0.049, 2.351 (1.313)	-0.089, -0.397 (0.124) **
Ethnicity - Black	-0.009, -0.497 (1.565)	0.062, 0.332 (0.148) *
Ethnicity - Hispanic	0.060, 3.975 (1.774) *	-0.045, -0.277 (0.168)
Ethnicity - Mixed/ Other	0.034, 1.162 (0.926)	-0.015, -0.048 (0.087)
Sample	0.014, 0.406 (1.175)	-0.107, -0.283 (0.111) *
Unemployment	0.053, 2.381 (1.210)*	-0.095, -0.394 (0.114) **
SES	-0.340, -3.388 (0.279) ***	0.378, 0.349 (0.026) ***
BMI	0.086, 0.182 (0.058) **	-0.067, -0.013 (0.005) *
Health Condition	0.190, 5.403 (0.792) ***	-0.085, -0.222 (0.075) **
Antidepressant use	0.135, 4.393 0.893) ***	-0.100, -0.301 (0.084) ***
Supplement use	-0.005, -0.075 (0.427)	0.022, 0.032 (0.040)
Food Allergy	0.037, 1.301 (0.948)	0.017, 0.056 (0.090)
Vegetarian	0.019, 0.969 (1.376)	-0.008, -0.039 (0.130)
Alcohol	0.030, 0.323 (0.305)	-0.025, -0.025 (0.029)
Smoking	0.038, 1.758 (1.244)	0.012, 0.050 (0.118)
R^2	0.265***	0.234***
F change *(df)*	21.901 (18,1092)	18.578 (18,1092)

Note. B = standardized regression coefficient, b= unstandardized regression coefficient; BMI = body mass index; FV = fruits and vegetables; SE = standard error; SES = socioeconomic status; continuous measures were all centered. *$p < .05$; **$p < .01$; ***$p < .001$

Supplementary Table 5

Hierarchical Regression Model 3 testing for Interactions among Health Behaviors to Predict Depressive Symptoms and Flourishing (n = 1111).

Model 3- adding interactions	Depressive Symptoms B, b (SE)	Flourishing B, b (SE)
Intercept	n/a, 4.379 (4.331)	n/a, 4.663 (0.338) ***
Covariates		
Age	-0.112, -0.621 (0.199) **	0.073, 0.038 (0.019) *
Gender - Female	0.042, 1.187 (0.755)	0.071, 0.187 (0.071) **
Gender - Diverse	0.036, 3.653 (2.577)	0.029, 0.269 (0.242)
Ethnicity - Asian	0.037, 1.781 (1.231)	-0.067, -0.301 (0.116) **
Ethnicity - Black	0.009, 0.534 (1.455)	0.044, 0.232 (0.137)
Ethnicity - Hispanic	0.054, 3.618 (1.644) *	-0.042, -0.260 (0.155)
Ethnicity - Mixed/ Other	0.022, 0.764 (0.868)	-0.008, -0.026 (0.082)
Sample	0.022, 0.621 (1.114)	-0.088, -0.233 (0.105) *
Unemployment	0.034, 1.525 (1.124)	-0.070, -0.290 (0.106) **
SES	-0.225, -2.238 (0.271) ***	0.264, 0.244 (0.026) *
BMI	0.036, 0.076 (0.055)	-0.003, -0.001 (0.005)
Health Condition	0.140, 3.983 (0.742) ***	-0.037, -0.096 (0.070)
Antidepressant use	0.111, 3.606 (0.834) ***	-0.063, -0.189 (0.078) *
Supplement use	0.003, 0.056 (0.400)	0.004, 0.006 (0.038)
Food Allergy	0.029, 1.009 (0.886)	0.018, 0.057 (0.083)
Vegetarian	0.015, 0.760 (1.287)	-0.009, -0.041 (0.121)
Alcohol	0.002, 0.025 (0.286)	0.008, 0.008 (0.027)
Smoking	0.012, 0.542 (1.177)	0.042, 0.180 (0.111)
Health Behaviors		
Sleep Quantity	-0.424, -4.055 (1.043) ***	-0.060, -0.053 (0.046)
Sleep Quantity Quadratic	0.395, 0.229 (0.065) ***	-0.087, -0.017 (0.006) **
Sleep Quality	-0.348, -4.502 (1.718) **	0.254, 0.304 (0.162)
Physical Activity	-0.075, -0.503 (0.181) **	0.143, 0.088 (0.017) ***
Raw FV	0.121, 0.895 (1.065)	-0.226, -0.155 (0.101)
Raw FV Quadratic	-	-0.056, -0.008 (0.006)
Processed FV	0.045, 0.562 (0.330)	-0.046, -0.054 (0.031)
Fast Food	0.019, 0.646 (0.919)	-0.048, -0.149 (0.086)
Sweets	0.045, 0.623 (0.360)	0.001, 0.001 (0.034)
Soda	0.008, 0.108 (0.340)	-0.021, -0.025 (0.032)
Interactions		
Sleep Quantity X Sleep Quality	0.060, 0.095 (0.227)	0.042, 0.006 (0.021)
Sleep Quantity X Activity	-0.018, -0.077 (0.133)	-0.010, -0.004 (0.013)
Sleep Quantity X Raw FV	-0.196, -0.190 (0.147)	0.374, 0.034 (0.014) *
Sleep Quality X Activity	0.018, 0.115 (0.170)	-0.046, -0.027 (0.016)
Sleep Quality X Raw FV	-0.010, -0.071 (0.192)	-0.023, -0.015 (0.018)
Raw FV X Activity	0.057, 0.186 (0.093) *	-0.065, -0.020 (0.010) *
R^2 change for Interactions	0.004	0.010**
F change (*df*) for Interactions	1.278 (6,1077)	2.923 (6,1076)
Cross validated R^2	0.342 (0.070)	0.308 (0.066)
Overall R^2	0.384	0.364

Note. b= unstandardized regression coefficient; BMI = body mass index; FV = fruits and vegetables; SE = standard error; SES = socioeconomic status; continuous measures were all centered. *$p < .05$; **$p < .01$; ***$p < .001$

Supplementary code

Adam Bartonicek

30/06/2020

```
library(tidyverse)
library(haven)
library(labelled)
library(janitor)
library(broom)
library(caret)
library(cowplot)

theme_set(theme_bw() + theme(panel.grid.major = element_blank(),
                             panel.grid.minor = element_blank()))
```

Data pre-processing

I first read in the data & changed all variable to lowercase to make working with them easier. After that, I selected participants who passed the inclusion checks, selected the relevant variables, changed some variables to factors (R's categorical variables), and recoded the ethnicity category into six levels (Asian, Black, Hispanic, White, and Other).

```
data_shay <- read_sav('2018-2019_Lifestyle_of_Young_Adults_Survey.sav')

data_shay_pre1 <- data_shay %>%
  clean_names()

data_shay_pre2 <- data_shay_pre1 %>%
  filter(included == 1) %>%
  select(age_c, gender, ethnicity_cat, sample, unemployed,
         ses_c, bmi_c, condition, allergy, vegetarian,
         alcohol_daily_c, medmood, vitsup, regsmoke,
         sleep_quantity_c = sleeep_quantity_c, sleep_quality_c = sleeep_quality_c,
         raw_fv_c, activity_c,
         cooked_fv_c, fastfood_daily_c, sweets_daily_c,
         soda_daily_c, flourishing_c, cesd_c) %>%
  mutate_at(vars(gender, ethnicity_cat, unemployed, condition, allergy,
                 vegetarian, medmood), ~ to_factor(.x)) %>%
  mutate_at(vars(sample, regsmoke), ~ factor(.x)) %>%
  mutate(ethnicity_cat = fct_other(ethnicity_cat,
                                   keep = c('Asian', 'Black', 'Hispanic', 'White'))
         %>% relevel(., ref = 'White'))
```

```r
set.seed(123)

seeds <- vector(mode = "list", length = 101)
for(i in 1:101) seeds[[i]]<- sample.int(n=1000)

hlthbehaviors <- c('sleep_quantity_c', 'sleep_quality_c', 'raw_fv_c', 'activity_c')

control <- trainControl(method = 'repeatedcv', number = 10, repeats = 10, seeds = seeds)

pred_combns <- tibble(x1 = hlthbehaviors, x2 = hlthbehaviors) %>%
  expand(x1, x2) %>%
  filter(x1 != x2) %>%
  rowwise() %>%
        mutate(id = paste0(sort(c(x1, x2)), collapse = " ")) %>%
    distinct(id, .keep_all = TRUE) %>%
    select(-id)

ints <- c('+ I(sleep_quantity_c ^ 2) + I(raw_fv_c ^ 2)', paste0('+ I(sleep_quantity_c ^ 2) + I(raw_fv_c

labels <- c('Baseline (health habits + quad)', 'Int. Activity vs Raw FV', 'Int. Activity vs Sleep qual.
  'Int. Activity vs Sleep quant.', 'Int. Raw FV vs Sleep qual.', 'Int. Raw FV vs Sleep quant.',
  'Int. Sleep qual. vs Sleep quant.')

flourishing_ints <- map(ints, ~ train(
  as.formula(paste0('flourishing_c ~ . - cesd_c', paste0(.x))),
  method = 'lm',
  data = data_shay_pre2,
  trControl = control
))

cesd_ints <- map(ints, ~ train(
  as.formula(paste0('cesd_c ~ . - flourishing_c', paste0(.x))),
  method = 'lm',
  data = data_shay_pre2,
  trControl = control
))

flourishing_results_df <- map(flourishing_ints, ~ pluck(.x, 'resample')) %>%
  bind_rows() %>%
  mutate(model = rep(labels, each = 100))

cesd_results_df <- map(cesd_ints, ~ pluck(.x, 'resample')) %>%
  bind_rows() %>%
  mutate(model = rep(labels, each = 100))

ggplot(flourishing_results_df, aes(model, Rsquared)) +
  geom_jitter(height = 0, width = 0.1, size = 1, col = 'grey40') +
  geom_boxplot(alpha = 0.75, fill = 'grey80', col = 'grey40', outlier.color = NA) +
  scale_x_discrete(guide = guide_axis(n.dodge = 2)) +
  labs(x = 'Model', y = 'Cross-validated R2 - Flourishing')
```

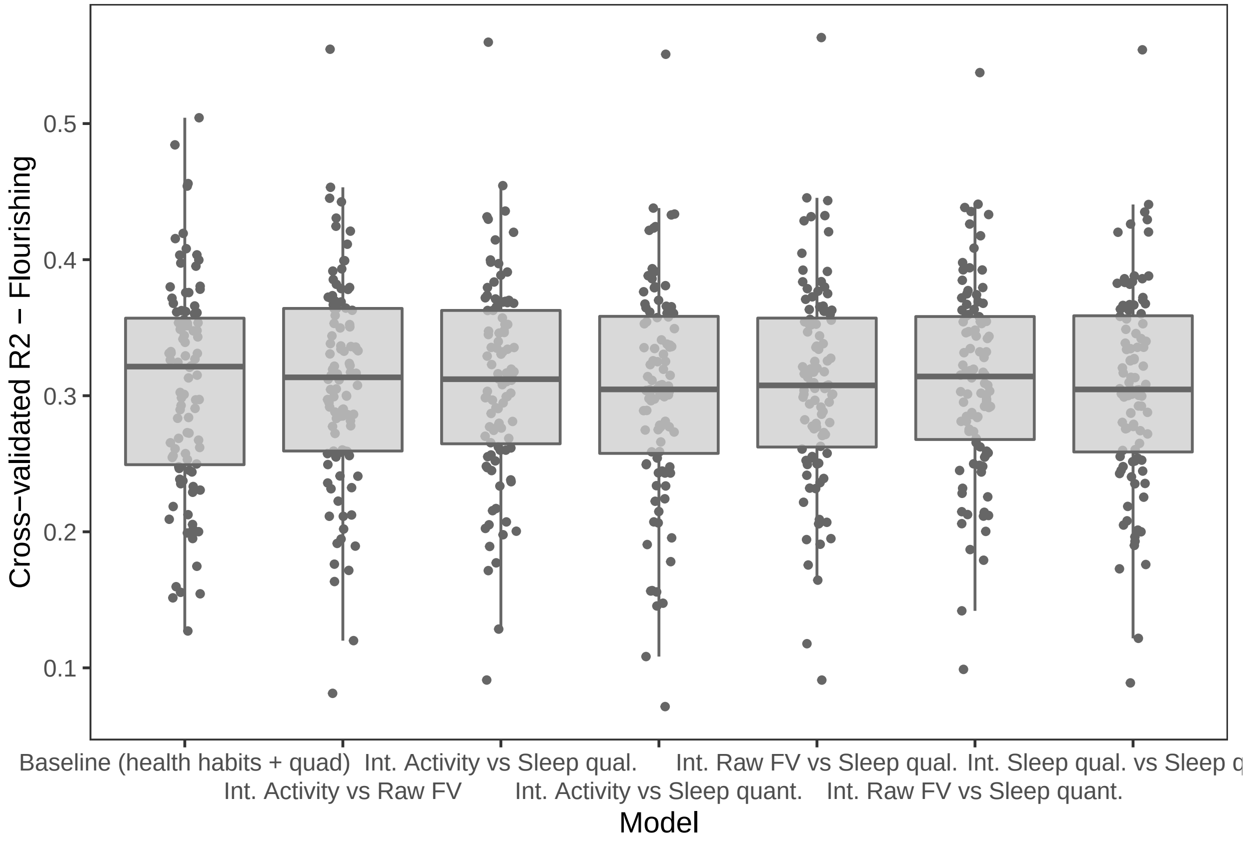


```
ggplot(cesd_results_df, aes(model, Rsquared)) +
  geom_jitter(height = 0, width = 0.1, size = 1, col = 'grey40') +
  geom_boxplot(alpha = 0.75, fill = 'grey80', col = 'grey40', outlier.color = NA) +
  scale_x_discrete(guide = guide_axis(n.dodge = 2)) +
  labs(x = 'Model', y = 'Cross-validated R2 - Depressive Symptoms')
```

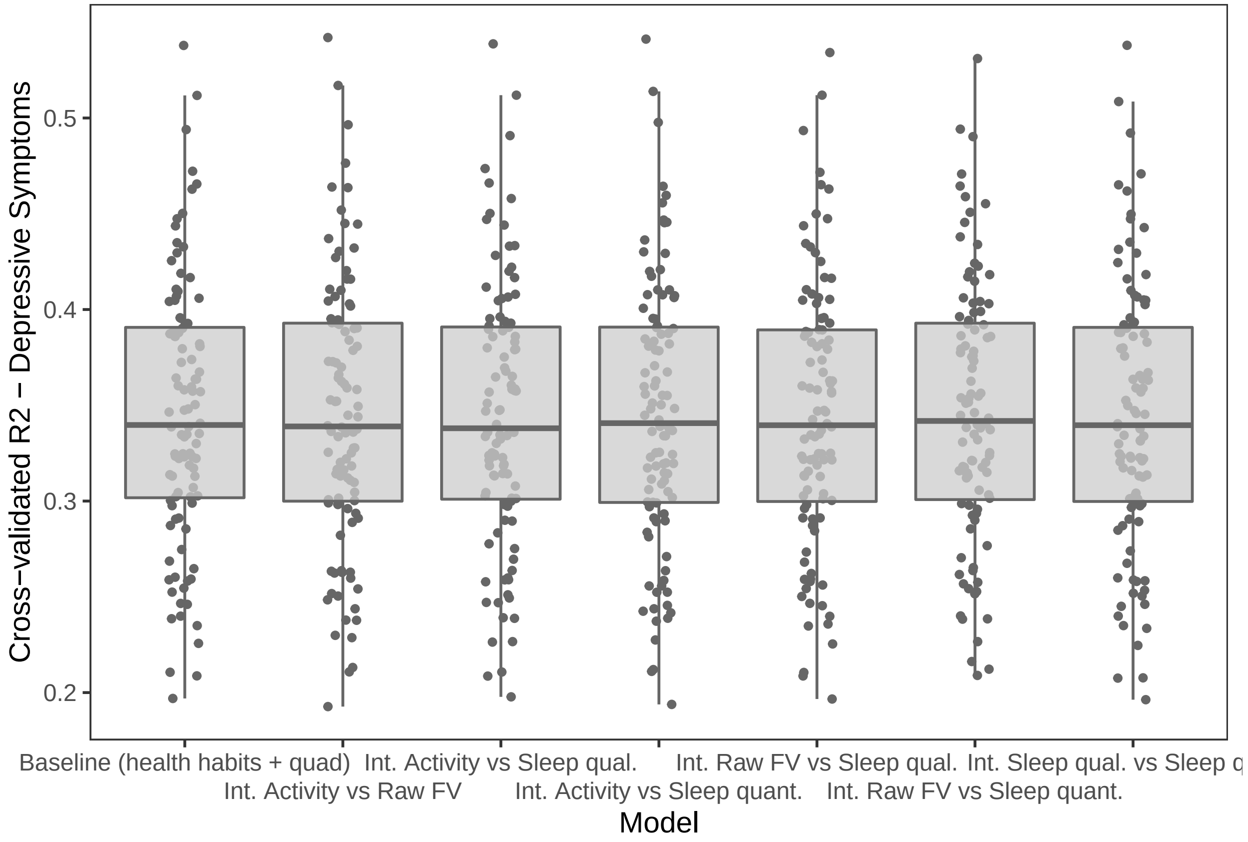


```
flourishing_results_df %>%
  group_by(model) %>%
  summarize(RMSE_mean = mean(RMSE),
            RMSE_sd = sd(RMSE),
            R2_mean = mean(Rsquared),
            R2_sd = sd(Rsquared)) %>%
  select(Model = model, `Mean RMSE` = RMSE_mean,
         `RMSE std. dev` = RMSE_sd, `Mean R2` = R2_mean,
         `R2 std. dev` = R2_sd) %>%
  knitr::kable(caption = 'Cross-validated performance across models predicting Flourishing')
```

Table 1: Cross-validated performance across models predicting Flourishing

Model	Mean RMSE	RMSE std. dev	Mean R2	R2 std. dev
Baseline (health habits + quad)	1.009670	0.0843666	0.3066713	0.0771294
Int. Activity vs Raw FV	1.009060	0.0882026	0.3099076	0.0759209
Int. Activity vs Sleep qual.	1.007366	0.0828656	0.3105251	0.0728124
Int. Activity vs Sleep quant.	1.017013	0.0973331	0.3028950	0.0779807
Int. Raw FV vs Sleep qual.	1.009393	0.0842756	0.3082801	0.0735747
Int. Raw FV vs Sleep quant.	1.005565	0.0786686	0.3112747	0.0703279
Int. Sleep qual. vs Sleep quant.	1.010694	0.0842497	0.3064232	0.0728548

```r
cesd_results_df %>%
  group_by(model) %>%
  summarize(RMSE_mean = mean(RMSE),
            RMSE_sd = sd(RMSE),
            R2_mean = mean(Rsquared),
            R2_sd = sd(Rsquared)) %>%
  select(Model = model, `Mean RMSE` = RMSE_mean,
         `RMSE std. dev` = RMSE_sd, `Mean R2` = R2_mean,
         `R2 std. dev` = R2_sd) %>%
  knitr::kable(caption = 'Cross-validated performance across models predicting Depressive Symptoms')
```

Table 2: Cross-validated performance across models predicting Depressive Symptoms

Model	Mean RMSE	RMSE std. dev	Mean R2	R2 std. dev
Baseline (health habits + quad)	10.57248	0.6288402	0.3454029	0.0697365
Int. Activity vs Raw FV	10.57713	0.6426332	0.3453489	0.0707459
Int. Activity vs Sleep qual.	10.58015	0.6291307	0.3445655	0.0698412
Int. Activity vs Sleep quant.	10.57985	0.6269172	0.3444854	0.0700546
Int. Raw FV vs Sleep qual.	10.58059	0.6301751	0.3445076	0.0698309
Int. Raw FV vs Sleep quant.	10.55389	0.6027675	0.3466046	0.0680471
Int. Sleep qual. vs Sleep quant.	10.58501	0.6320858	0.3441208	0.0699256

```r
flourishing_formula <- paste0('flourishing_c ~ . - cesd_c + I(sleep_quantity_c ^ 2) + I(raw_fv_c ^ 2) +
      paste(hlthbehaviors, collapse = ' + '), ') ^ 2')

cesd_formula <- paste0('cesd_c ~ . - flourishing_c + I(sleep_quantity_c ^ 2) + I(raw_fv_c ^ 2) + (',
      paste(hlthbehaviors, collapse = ' + '), ') ^ 2')

flourishing_train_all <- train(
  as.formula(flourishing_formula),
  method = 'lm',
  data = data_shay_pre2,
  trControl = control
)

cesd_train_all <- train(
  as.formula(cesd_formula),
  method = 'lm',
  data = data_shay_pre2,
  trControl = control
)

flourishing_train_all$resample %>%
  ggplot(aes(x = 'Model with all quadratic terms & interactions', y = Rsquared)) +
  geom_jitter(height = 0, width = 0.1, size = 1, col = 'grey40') +
  geom_boxplot(alpha = 0.75, fill = 'grey80', col = 'grey40', outlier.color = NA) +
  scale_x_discrete(guide = guide_axis(n.dodge = 2)) +
  labs(x = '', y = 'Cross-validated R2 - Flourishing')
```

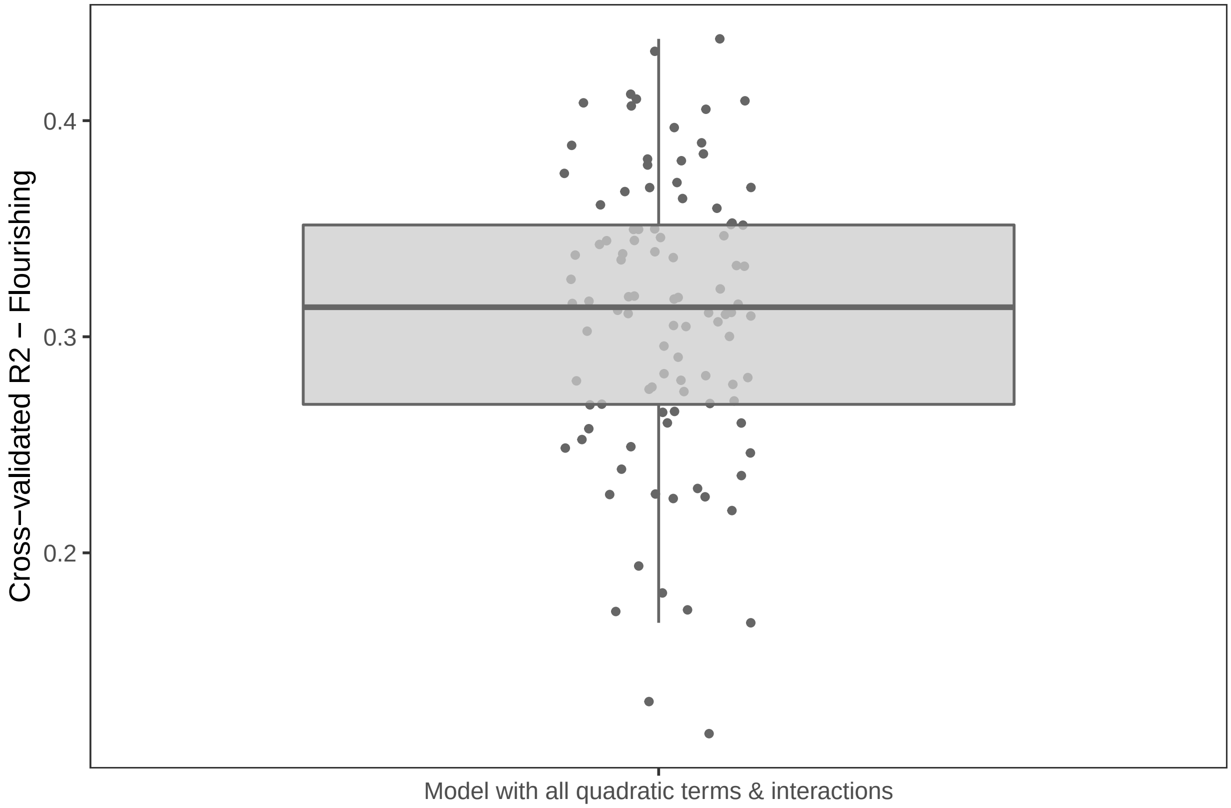


```r
cesd_train_all$resample %>%
  ggplot(aes(x = 'Model with all quadratic terms & interactions', y = Rsquared)) +
  geom_jitter(height = 0, width = 0.1, size = 1, col = 'grey40') +
  geom_boxplot(alpha = 0.75, fill = 'grey80', col = 'grey40', outlier.color = NA) +
  scale_x_discrete(guide = guide_axis(n.dodge = 2)) +
  labs(x = '', y = 'Cross-validated R2 - Depressive symptoms')
```

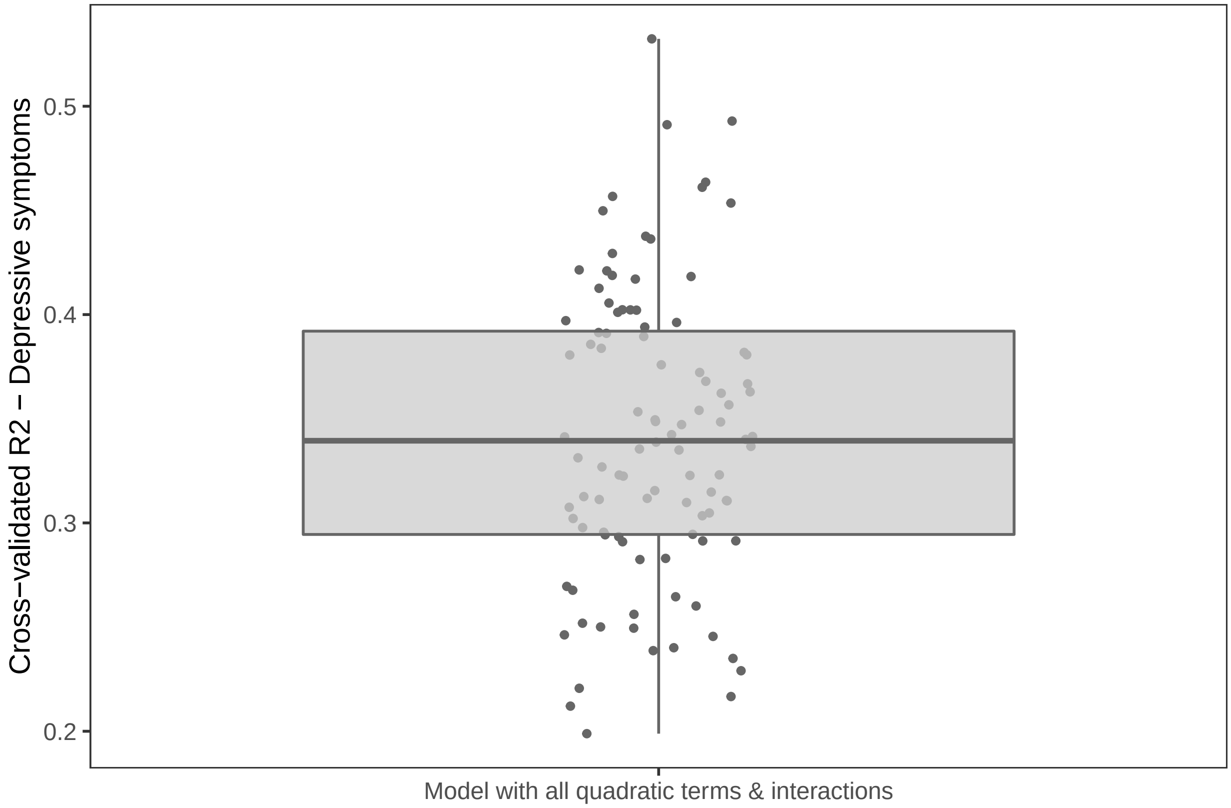


```
flourishing_train_all$resample %>%
  summarize(RMSE_mean = mean(RMSE),
            RMSE_sd = sd(RMSE),
            R2_mean = mean(Rsquared),
            R2_sd = sd(Rsquared)) %>%
  select(`Mean RMSE` = RMSE_mean,
         `RMSE std. dev` = RMSE_sd, `Mean R2` = R2_mean,
         `R2 std. dev` = R2_sd) %>%
  knitr::kable(caption = 'Cross-validated performance of the model with all quadratic terms & interacti
```

Table 3: Cross-validated performance of the model with all quadratic terms & interactions, predicting Flourishing

Mean RMSE	RMSE std. dev	Mean R2	R2 std. dev
1.009659	0.081944	0.3084138	0.0660169

```
cesd_train_all$resample %>%
  summarize(RMSE_mean = mean(RMSE),
            RMSE_sd = sd(RMSE),
            R2_mean = mean(Rsquared),
            R2_sd = sd(Rsquared)) %>%
  select(`Mean RMSE` = RMSE_mean,
         `RMSE std. dev` = RMSE_sd, `Mean R2` = R2_mean,
```

```
        `R2 std. dev` = R2_sd) %>%
  knitr::kable(caption = 'Cross-validated performance of the model with all quadratic terms & interacti
```

Table 4: Cross-validated performance of the model with all quadratic terms & interactions, predicting Depressive symptoms

Mean RMSE	RMSE std. dev	Mean R2	R2 std. dev
10.60009	0.6197262	0.3420665	0.0698725

```
marginal_predictions <- function(fit = NULL, vars = c(NULL), n = 1000) {

  data <- fit$model

  if (length(vars) == 2) {

  var1_min <- data %>% pluck(vars[1]) %>% min()
  var1_max <- data %>% pluck(vars[1]) %>% max()
  var1_range <- seq(var1_min, var1_max, length.out = n)
  var2_quants <- data %>% pluck(vars[2]) %>% quantile(., c(0.025, 0.5, 0.975))

  grid <- expand.grid(var1_range, var2_quants) %>% as_tibble()

  empty_tibble <- data %>%
    mutate_if(is.numeric, ~ 0) %>%
    mutate_if(is.factor, ~ levels(.x)[1]) %>%
    slice(1:n) %>%
    select(- vars)

  empty_tibble <- bind_rows(list(empty_tibble, empty_tibble, empty_tibble))

  marginal_tibble <- grid %>%
    bind_cols(empty_tibble)

  } else {

  var_min <- data %>% pluck(vars) %>% min()
  var_max <- data %>% pluck(vars) %>% max()
  var_range <- seq(var_min, var_max, length.out = n)

  marginal_tibble <- data %>%
    mutate_if(is.numeric, ~ 0) %>%
    mutate_if(is.factor, ~ levels(.x)[1]) %>%
    slice(1:n) %>%
    mutate(!!vars := var_range)

  }

  preds <- predict(fit, newdata = marginal_tibble, interval = 'confidence')

  if (length(vars) == 2) {
```

```r
  as_tibble(preds) %>%
    mutate(predictor1 := !!vars[1],
           predictor1_value := var1_range,
           predictor2 := !!vars[2],
           predictor2_value := var2_range) %>%
      select(predictor1, predictor1_value,
             predictor2, predictor2_value,
             predicted = fit, lwr, upr)

  } else {

  as_tibble(preds) %>%
    mutate(predictor := !!vars[1],
           predictor_value := var_range)%>%
    select(predictor, predictor_value, predicted = fit, lwr, upr)

}}
```

```r
fit1_flourishing <- lm(flourishing_c ~ . - cesd_c + I(sleep_quantity_c ^ 2) + I(raw_fv_c ^ 2),
                       data = data_shay_pre2)
fit2_cesd <- lm(cesd_c ~ . - flourishing_c + I(sleep_quantity_c ^ 2) + I(raw_fv_c ^ 2),
                       data = data_shay_pre2)

predictor <- c('sleep_quantity_c', 'sleep_quality_c',
               'activity_c', 'raw_fv_c')

label <- c('Sleep quantity', 'Sleep quality',
           'Physical activity', 'Raw fruit and vegetables')

label_levs <- label

predlabel <- tibble(predictor, label)

flourishing_offset <- mean(data_shay$FLOURISHING_MEAN, na.rm = TRUE)
cesd_offset <- mean(data_shay$CESD_SUM, na.rm = TRUE)
sleepqual_offset <- mean(data_shay$SLEEPQUAL, na.rm = TRUE)
sleepquant_offset <- mean(data_shay$SLEEP, na.rm = TRUE)
activity_offset <- mean(data_shay$ACTIVITY, na.rm = TRUE)
rawfv_offset <- mean(data_shay$RAWFV_DAILY, na.rm = TRUE)

flourishing_plot_data <- map(predictor,
                             ~ marginal_predictions(fit1_flourishing,
                                                    var = .x)) %>%
  bind_rows() %>%
  left_join(predlabel) %>%
  mutate(label = factor(label, levels = label_levs),
         predicted = predicted + flourishing_offset,
         upr = upr + flourishing_offset,
         lwr = lwr + flourishing_offset,
         predictor_value = case_when(
           predictor == 'sleep_quantity_c' ~ predictor_value + sleepquant_offset,
           predictor == 'sleep_quality_c' ~ predictor_value + sleepqual_offset,
           predictor == 'activity_c' ~ predictor_value + activity_offset,
```

```r
          predictor == 'raw_fv_c' ~ predictor_value + rawfv_offset
        ))

cesd_plot_data <- map(predictor,
                      ~ marginal_predictions(fit2_cesd,
                                             var = .x)) %>%
  bind_rows() %>%
  left_join(predlabel) %>%
  mutate(label = factor(label, levels = label_levs),
         predicted = predicted + cesd_offset,
         upr = upr + cesd_offset,
         lwr = lwr + cesd_offset,
         predictor_value = case_when(
           predictor == 'sleep_quantity_c' ~ predictor_value + sleepquant_offset,
           predictor == 'sleep_quality_c' ~ predictor_value + sleepqual_offset,
           predictor == 'activity_c' ~ predictor_value + activity_offset,
           predictor == 'raw_fv_c' ~ predictor_value + rawfv_offset
           ))

cesd_plot_data %>%
  group_by(predictor) %>%
  filter(predicted == max(predicted) | predicted == min(predicted))
```

```
## # A tibble: 8 x 6
## # Groups:   predictor [4]
##   predictor        predictor_value predicted   lwr   upr label
##   <chr>                      <dbl>     <dbl> <dbl> <dbl> <fct>
## 1 sleep_quantity_c         9.74       13.5  11.0  16.0  Sleep quantity
## 2 sleep_quantity_c        20.0        33.0  15.7  50.2  Sleep quantity
## 3 sleep_quality_c          0.00252    20.9  18.3  23.4  Sleep quality
## 4 sleep_quality_c          4.00        5.73  2.98  8.48 Sleep quality
## 5 activity_c              -0.0100     16.2  13.6  18.8  Physical activity
## 6 activity_c               6.99       12.6  10.1  15.1  Physical activity
## 7 raw_fv_c                 5.47       13.8  11.0  16.5  Raw fruit and vegetabl~
## 8 raw_fv_c                14.0        19.5   7.57 31.4  Raw fruit and vegetabl~
```

```r
flourishing_plot_data %>%
  group_by(predictor) %>%
  filter(predicted == max(predicted) | predicted == min(predicted))
```

```
## # A tibble: 8 x 6
## # Groups:   predictor [4]
##   predictor        predictor_value predicted   lwr   upr label
##   <chr>                      <dbl>     <dbl> <dbl> <dbl> <fct>
## 1 sleep_quantity_c         8.00         5.21  4.99  5.42 Sleep quantity
## 2 sleep_quantity_c        20.0          3.62  1.98  5.25 Sleep quantity
## 3 sleep_quality_c          0.00252      4.64  4.40  4.88 Sleep quality
## 4 sleep_quality_c          4.00         6.02  5.76  6.28 Sleep quality
## 5 activity_c              -0.0100       4.93  4.69  5.18 Physical activity
## 6 activity_c               6.99         5.57  5.33  5.81 Physical activity
## 7 raw_fv_c                 4.76         5.30  5.06  5.55 Raw fruit and vegetabl~
## 8 raw_fv_c                14.0          4.14  3.02  5.27 Raw fruit and vegetabl~
```

```r
flourishing_plot_data <- flourishing_plot_data %>%
  filter(predictor != 'sleep_quantity_c' | predictor_value < 6 + sleepquant_offset) %>%
  filter(predictor != 'raw_fv_c' | predictor_value < 9 + rawfv_offset)

cesd_plot_data <- cesd_plot_data %>%
  filter(predictor != 'sleep_quantity_c' | predictor_value < 6 + sleepquant_offset) %>%
  filter(predictor != 'raw_fv_c' | predictor_value < 9 + rawfv_offset)

flourishing_max_y <- max(flourishing_plot_data$upr)
flourishing_min_y <- min(flourishing_plot_data$lwr)
cesd_max_y <- max(cesd_plot_data$upr)
cesd_min_y <- min(cesd_plot_data$lwr)

breaks_fun <- function(x) {
  if (max(x) > 10) {
    seq(0, 16, 2)
  } else {
    seq(0, 10, 1)
  }
}

flourishing_plot <- ggplot(flourishing_plot_data, aes(x = predictor_value, y = predicted,
                            ymin = lwr, ymax = upr)) +
  geom_ribbon(fill = 'grey90') +
  geom_line() +
  facet_wrap(~ label, ncol = 1, scales = 'free') +
  scale_x_continuous(breaks = breaks_fun) +
  scale_y_continuous(breaks = seq(3, 7, by = 0.5), limits = c(flourishing_min_y, flourishing_max_y)) +
  labs(x = NULL, y = 'Predicted Flourshing') +
  theme_bw() +
  theme(panel.grid.major = element_blank(),
        panel.grid.minor = element_blank())

cesd_plot <- ggplot(cesd_plot_data, aes(x = predictor_value, y = predicted,
                            ymin = lwr, ymax = upr)) +
  geom_ribbon(fill = 'grey90') +
  geom_line() +
  facet_wrap(~ label, ncol = 1, scales = 'free') +
  scale_x_continuous(breaks = breaks_fun) +
  ylim(cesd_min_y, cesd_max_y) +
  labs(x = NULL, y = 'Predicted Depressive Symptoms') +
  theme_bw() +
  theme(panel.grid.major = element_blank(),
        panel.grid.minor = element_blank())

plot_grid(cesd_plot, flourishing_plot)
```

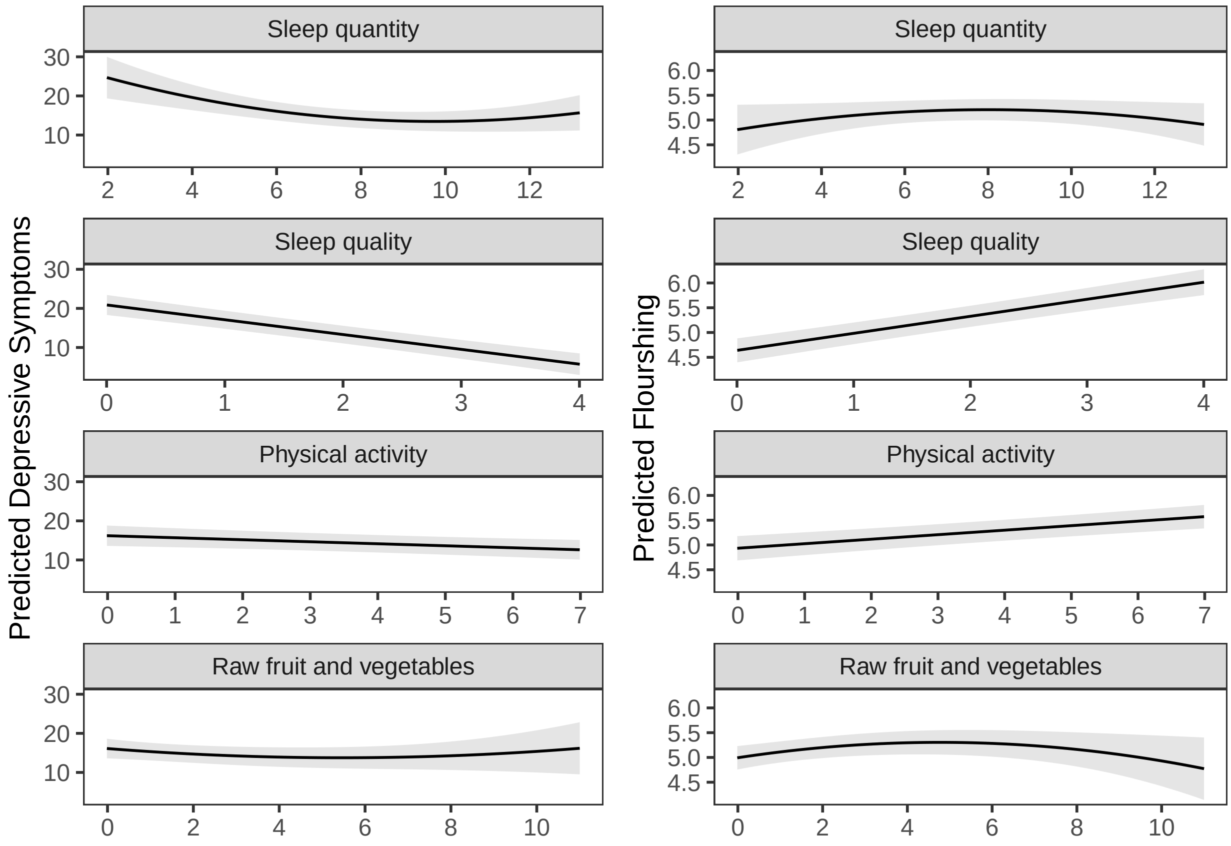


```r
ggsave('figure_lifestyle.png', width = 180, height = 280, units = 'mm')
```

```r
fit1_flourishing %>%
  tidy() %>%
  knitr::kable(caption = 'Model predicting flourishing')
```

Table 5: Model predicting flourishing

term	estimate	std.error	statistic	p.value
(Intercept)	0.1962744	0.1084224	1.8102748	0.0705305
age_c	0.0377934	0.0187875	2.0116230	0.0445073
genderFemale	0.1899284	0.0712753	2.6647142	0.0078200
genderGender Diverse	0.2567945	0.2424706	1.0590747	0.2898021
ethnicity_catAsian	-0.3037168	0.1164165	-2.6088802	0.0092092
ethnicity_catBlack	0.2276921	0.1374320	1.6567616	0.0978576
ethnicity_catHispanic	-0.2748113	0.1550834	-1.7720229	0.0766721
ethnicity_catOther	-0.0237953	0.0818443	-0.2907387	0.7713069
sample1	-0.2556121	0.1050430	-2.4334043	0.0151187
unemployedUnemployed	-0.2922670	0.1060942	-2.7547876	0.0059715
ses_c	0.2438261	0.0255655	9.5373175	0.0000000
bmi_c	-0.0008041	0.0052027	-0.1545510	0.8772041
conditionHas health condition	-0.1003219	0.0700860	-1.4314115	0.1526011
allergyHas Food Allergy	0.0643986	0.0835385	0.7708849	0.4409435
vegetarianEats no meats	-0.0047516	0.1214557	-0.0391224	0.9688000

term	estimate	std.error	statistic	p.value
alcohol_daily_c	0.0079248	0.0269539	0.2940137	0.7688038
medmoodYES	-0.1979565	0.0787034	-2.5152221	0.0120397
vitsup	0.0115840	0.0377931	0.3065103	0.7592751
regsmoke1	0.1661125	0.1110521	1.4958071	0.1349955
sleep_quantity_c	0.0180673	0.0246726	0.7322811	0.4641556
sleep_quality_c	0.3437674	0.0337698	10.1797209	0.0000000
raw_fv_c	0.0751033	0.0266808	2.8148844	0.0049680
activity_c	0.0910143	0.0170438	5.3400149	0.0000001
cooked_fv_c	-0.0602906	0.0312355	-1.9301961	0.0538437
fastfood_daily_c	-0.1508405	0.0864326	-1.7451802	0.0812374
sweets_daily_c	-0.0041390	0.0339709	-0.1218406	0.9030479
soda_daily_c	-0.0304593	0.0319103	-0.9545298	0.3400287
I(sleep_quantity_c^2)	-0.0111000	0.0054978	-2.0190014	0.0437333
I(raw_fv_c^2)	-0.0136575	0.0051440	-2.6550302	0.0080466

```
fit2_cesd %>%
  tidy() %>%
  knitr::kable(caption = 'Model predicting Depressive Symptoms')
```

Table 6: Model predicting Depressive Symptoms

term	estimate	std.error	statistic	p.value
(Intercept)	-4.6904110	1.1467750	-4.0900885	0.0000463
age_c	-0.6338210	0.1987142	-3.1896115	0.0014656
genderFemale	1.1244499	0.7538732	1.4915636	0.1361051
genderGender Diverse	3.8410714	2.5645919	1.4977320	0.1344945
ethnicity_catAsian	1.7423461	1.2313281	1.4150137	0.1573522
ethnicity_catBlack	0.5374954	1.4536071	0.3697667	0.7116287
ethnicity_catHispanic	3.6755124	1.6403039	2.2407508	0.0252445
ethnicity_catOther	0.7433974	0.8656603	0.8587634	0.3906612
sample1	0.7260835	1.1110310	0.6535223	0.5135585
unemployedUnemployed	1.5293597	1.1221495	1.3628841	0.1732026
ses_c	-2.2464002	0.2704039	-8.3075718	0.0000000
bmi_c	0.0797207	0.0550287	1.4487113	0.1477080
conditionHas health condition	3.9571494	0.7412938	5.3381662	0.0000001
allergyHas Food Allergy	0.9728219	0.8835797	1.1010007	0.2711412
vegetarianEats no meats	0.5650534	1.2846267	0.4398581	0.6601277
alcohol_daily_c	0.0091703	0.2850890	0.0321664	0.9743453
medmoodYES	3.6028094	0.8324394	4.3280140	0.0000164
vitsup	0.0373313	0.3997349	0.0933902	0.9256109
regsmoke1	0.5422125	1.1745890	0.4616189	0.6444474
sleep_quantity_c	-0.9505182	0.2609601	-3.6423892	0.0002829
sleep_quality_c	-3.7852337	0.3571807	-10.5975329	0.0000000
raw_fv_c	-0.5430293	0.2822003	-1.9242689	0.0545829
activity_c	-0.5136432	0.1802712	-2.8492799	0.0044649
cooked_fv_c	0.6292102	0.3303753	1.9045319	0.0571059
fastfood_daily_c	0.5463396	0.9141904	0.5976212	0.5502177
sweets_daily_c	0.6623351	0.3593070	1.8433684	0.0655486
soda_daily_c	0.1376289	0.3375124	0.4077744	0.6835199
I(sleep_quantity_c^2)	0.1859461	0.0581495	3.1977223	0.0014253
I(raw_fv_c^2)	0.0787783	0.0544076	1.4479286	0.1479267
